# Anti-biofilm activity of an exopolysaccharide from a sponge-associated strain of *Bacillus licheniformis*

**DOI:** 10.1186/1475-2859-10-74

**Published:** 2011-09-27

**Authors:** SM Abu Sayem, Emiliano Manzo, Letizia Ciavatta, Annabella Tramice, Angela Cordone, Anna Zanfardino, Maurilio De Felice, Mario Varcamonti

**Affiliations:** 1 Department of Structural and Functional Biology, University of Naples Federico II, Naples, Italy; 2 Institute of Biomolecular chemistry, National Center for Research, Naples, Italy; 3 Department of Genetic Engineering and Biotechnology, Shahjalal University of Science and Technology, Sylhet, Bangladesh

## Abstract

**Background:**

Secondary metabolites ranging from furanone to exo-polysaccharides have been suggested to have anti-biofilm activity in various recent studies. Among these, *Escherichia coli *group II capsular polysaccharides were shown to inhibit biofilm formation of a wide range of organisms and more recently marine *Vibrio *sp. were found to secrete complex exopolysaccharides having the potential for broad-spectrum biofilm inhibition and disruption.

**Results:**

In this study we report that a newly identified ca. 1800 kDa polysaccharide having simple monomeric units of α-D-galactopyranosyl-(1→2)-glycerol-phosphate exerts an anti-biofilm activity against a number of both pathogenic and non-pathogenic strains without bactericidal effects. This polysaccharide was extracted from a *Bacillus licheniformis *strain associated with the marine organism *Spongia officinalis*. The mechanism of action of this compound is most likely independent from quorum sensing, as its structure is unrelated to any of the so far known quorum sensing molecules. In our experiments we also found that treatment of abiotic surfaces with our polysaccharide reduced the initial adhesion and biofilm development of strains such as *Escherichia coli *PHL628 and *Pseudomonas fluorescens*.

**Conclusion:**

The polysaccharide isolated from sponge-associated *B. licheniformis *has several features that provide a tool for better exploration of novel anti-biofilm compounds. Inhibiting biofilm formation of a wide range of bacteria without affecting their growth appears to represent a special feature of the polysaccharide described in this report. Further research on such surface-active compounds might help developing new classes of anti-biofilm molecules with broad spectrum activity and more in general will allow exploring of new functions for bacterial polysaccharides in the environment.

## Background

Most species of bacteria prefer biofilm as the most common means of growth in the environment and this kind of bacterial socialization has recently been described as a very successful form of life on earth [1]. Although they can have considerable advantages in terms of self-protection for the microbial community involved or to develop *in situ *bioremediation systems [2], biofilms have great negative impacts on the world's economy and pose serious problems to industry, marine transportation, public health and medicine due to increased resistance to antibiotics and chemical biocides, increased rates of genetic exchange, altered biodegradability and increased production of secondary metabolites [3-8]. Therefore, based on the above reasons, development of anti-biofilm strategies is of major concern.

The administration of antimicrobial agents and biocides in the local sites to some extent has been a useful approach to get rid of biofilms [9], but prolonged persistence of these compounds in the environment could induce toxicity towards non-target organisms and resistance among microorganisms within biofilms. This aspect has led to the development of more environment friendly compounds to combat with the issue. It has been found that many organisms in the marine areas maintain a clean surface. Most of the marine invertebrates have developed unique ways to combat against potential invaders, predators or other competitors [10] especially through the production of specific compounds toward biofilm-forming microorganisms [11]. Nowadays, it is hypothesized that bioactive compounds previously thought to be produced from marine invertebrates might be produced by the associated microorganisms instead. Various natural compounds from marine bacteria, alone or in association with other invertebrates, are emerging as potential sources for novel metabolites [12] and have been screened to validate anti-biofilm activity. The quorum sensing antagonist (*5Z*)-4-bromo-5-(bromomethylene)-3-butyl-2(*5H*)-furanone (furanone) from the marine alga *Delisea pulchra *inhibits biofilm formation in *E. coli *without inhibiting its growth [13]. The metabolites of a marine actinomycete strain A66 inhibit biofilm formation by *Vibrio *in marine ecosystem [12]. Extracts from coral associated *Bacillus horikoshii *[14] and actinomycetes [15] inhibit biofilm formation of *Streptococcus pyogenes*. The exoproducts of marine *Pseudoalteromonas *impair biofilm formation by a wide range of pathogenic strains [16]. Most recently, exo-polysaccharides from the marine bacterium *Vibrio *sp. QY101 were shown to control biofilm-associated infections [17].

Compounds secreted or extracted from marine microorganisms having anti-biofilm activity range from furanone to complex polysaccharide. Although bacterial extracellular polysaccharides synthesized and secreted by a wide range of bacteria from various environments have been proven to be involved in pathogenicity [18], promotion of adherence to surfaces [19-21] and biofilm formation [22,23], recent findings suggest that some polysaccharides secreted from marine and non marine organisms also possess the ability to negatively regulate biofilm formation [17,24-27].

In this study, we show that an exo-polysaccharide purified from the culture supernatant of bacteria associated to a marine sponge (*Spongia officinalis*) is able to inhibit biofilm formation without affecting the growth of the tested strains. Phylogenetic analysis by 16S rRNA gene sequencing identified the sponge-associated bacterium as *Bacillus licheniformis*. The mechanisms behind the anti-biofilm effect of the secreted exo-polysaccharide were preliminarily investigated.

## Results

### *Bacillus licheniformis *culture supernatant inhibits biofilm formation by *Escherichia coli *PHL628

**Figure 1:**
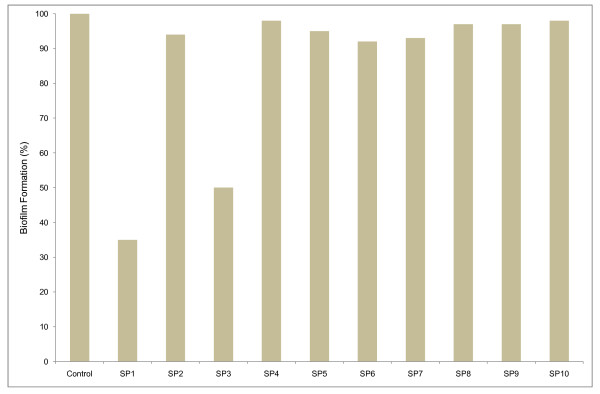
**Anti-biofilm activity of supernatants from different strains (SP1-SP10) associated with *Spongia officinalis***. Biofilms of *Escherichia coli *PHL628 were allowed to develop in the presence of supernatants (3% v/v) from marine sponge-associated isolates in 96 well microtiter well. The plate was incubated at 30°C for 36 h, followed by crystal violet staining and spectrophotometric absorbance measurements (OD_570_). The absorbance was used to calculate the "biofilm formation" on the *y axis. × axis *represents cell free supernatants from different *Spongia officinalis *isolates. The 100% is represented by *E. coli *PHL628 produced biofilm.

Starting from a *Spongia officinalis *sample, it has been possible to distinguish, among one hundred colonies of sponge-associated bacteria, ten different kinds in terms of shape, size and pigmentation. They were screened for production of bioactive anti-biofilm metabolites. One colony for each phenotype was grown till stationary phase and the filtered cell-free supernatants obtained were used at a concentration of 3% (v/v) against a stationary culture of the indicator strain *E. coli *PHL628 (Figure 1). Supernatants derived from strains SP1 and SP3 showed a strong anti-biofilm activity (65% and 50% reduction, respectively). SP1 was chosen to study the nature of the biofilm inhibition mechanism. Sequencing of the 16S RNA revealed that the SP1 gene showed 99% similarity with *Bacillus licheniformis*.

### Isolation and purification of active compounds

The active fraction of SP1 cell free supernatant was initially found to be of polysaccharidic composition. Preliminary spectroscopic investigations indicated the presence of a compound with a simple primary structure; the ^1^H and ^13^C NMR spectra suggested that the polymer was composed by a regular-repeating unit; the monosaccharide was identified as an acetylated O-methyl glycoside derivative and the compositional analysis was completed by the methylation data which indicated the presence of 4-substituted galactose; in fact the sample was methylated with iodomethane, hydrolized with 2 M triofluoroacetic acid (100°C, 2 h), the carbonyl was reduced by NaBD_4_, acetylated with acetic anhydride and pyridine, and analyzed by GC-MS. The molecular mass of the polysaccharidic molecule was estimated to be approximately 1800 kDa by gel filtration on a Sepharose CL6B. In TOCSY, DEPT-HSQC, and HSQC-TOCSY experiments, additional signals of a -CHO- and two -CH_2 _O- spin system proved the presence of not only a galactose residue but also of a glycerol residue (Gro); the relatively deshielded value for the glycerol methylene carbons at 65.6 and 65.4 ppm was consistent with a phosphate substitution at C1 of glycerol. ^31^P-NMR spectrum confirms the presence of a phosphodiester group.

The position of the phosphate group between the α-D-galactopyranosyl and the glycerol residue was unambiguously confirmed with 2D ^1^H ^31^P-HSQC experiments. In fact, correlations between the ^31^P resonance and H4 (3.827 ppm) of galactose were observed. This fact established the connectivity of the phosphate group to the respective carbon atoms. It follows that the repeating unit contains the phosphate diester fragment. Galactose was present as pyranose ring, as indicated by ^1^H- and ^13^C-NMR chemical shifts and by the HMBC spectrums that showed some typical intra-residual scalar connectivities between H/C (Table 1). The connection between galactose and glycerol into repeating unit was determined using HMBC and NOE effects. The anomeric site (99.47 and 5.071 ppm) of galactose presented long-range correlations with glycerol C2' (70.76 ppm) and H2' (4.120 ppm), and allowed the localization of galactose binding at C2' of glycerol. NOE contacts of anomeric proton at 5.071 ppm with the signal at 3.839 ppm (Gro H23', table 1) confirmed this hypothesis.

**Table 1:** ^1^H, ^13^C and ^31^P NMR chemical shift of polysaccharide(p.p.m). Spectra in D_2_O were measured at 27°C and referenced to internal sodium 3-(trimethylsilyl)-(2,2,3,3-^2^H_4_) propionate (δ_H _0.00), internal methanol (δ_C _49.00)and to external aq. 85% (v/v) phosphoric acid (δ_P _0.00)

Residue	Nucleus	1	2	3	4	5	6
→ 4)-α-D-Galp-(1 →							

	^1^H	5.071 ^H3Gro^ (3.7 Hz)^a^	3.690	3.784	*3.827*^C6,4Gal^	*3.917*^C3Gal^	3.671^H1Gal^

	^13^C	*99.47*^H1Gro^	69.37	69.95	*78.32*^H1Gal^ (7.8 Hz)^b^	70.19	*62.18*^H5Gal^

Gro-1-P-(O →							

	^1^H	*3.865 *^C4Gal ^-3.906	*4.120*^C3, 5Gro^	3.839-3.770			

	^13^C	65.63*^H1Gro^ (4 Hz)^a ^-65.41* (4.5)^a^	*70.76 ^H1Gal^* (7.9 Hz)^c^	67.15 (~2 Hz)^d^			

	^31^P	1.269					

*diastereotopic carbons; ^a 3^J _H1, H 2_; ^b 2^J _C-P _; ^c 3^J _C-P _; ^d 4^J _C-P_; in italics, the signals showing C-H long-range correlations with the positions in superscripts; underlined are the NOE contacts with positions in superscripts.

**Figure 2:**
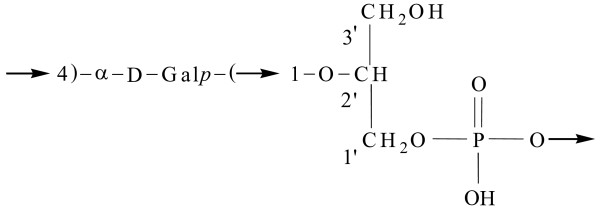
**Repeating unit of the bacterial polysaccharide having anti-biofilm activity**.

Thus, the polysaccharide is composed of α-D-galactopyranosyl-(1→2)-glycerol-phosphate monomeric units (Figure 2).

### The anti-biofilm activity does not result from reducing *E. coli *and *P. fluorescens *growth

**Figure 3:**
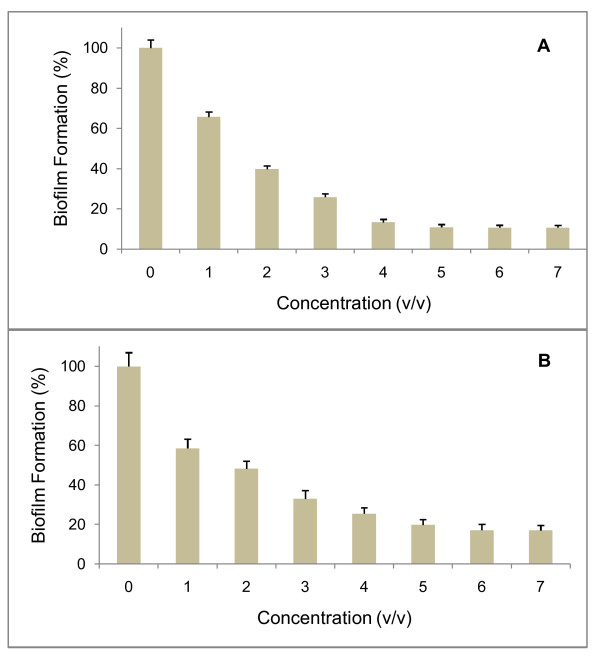
**Anti-biofilm activity is concentration-dependent**. Stationary cells of *E. coli *PHL628 (A) or *P. fluorescens *(B) were incubated along with the SP1 supernatant at different concentrations in 96-well microtiter plate. The plate was incubated at 30°C for 36 h, followed by crystal violet staining and spectrophotometric absorbance measurements (OD_570_). The ratio of biofilm absorbance/planktonic absorbance was calculated, and this value was used to calculate the "biofilm formation" on the *y axis. × axis *represents the concentration of supernatant used in the wells. Bars represent means ± standard errors for six replicates.

In order to check whether the anti-biofilm activity of the sponge-associated SP1 strain is dependent on the concentration used in the microtiter plate assay, the cell free supernatant from this strain was tested against biofilm formation by two organisms, *E. coli *PHL628 and *Pseudomonas fluorescens*. The results of Figure 3 clearly show that the anti-biofilm activity raises as the concentration of the supernatant increases. The anti-biofilm activity of the SP1 supernatant against the two test strains was comparable and perhaps slightly higher for *E. coli *PHL628, as in the presence of 5% (v/v) supernatant, inhibition was about 89% and 80% on biofilm formation by *E. coli *PHL 628 (Figure 3A) and *Pseudomonas fluorescens*, respectively (Figure 3B).

To evaluate whether the anti-biofilm effect of cell-free supernatant from sponge-associated *B. licheniformis *was related to reduction of growth rate of the target strains, growth curves of both strains were measured in presence and absence of 5% (v/v) supernatant. The resulting growth rates were found to be the same in the two conditions for both *E. coli *PHL628 (0.51 ± 0.02 h^-1^) and *P. fluorescens *(0.69 ± 0.02 h^-1^), clearly indicating that the supernatant has no bactericidal activity against the cells of biofilm-producing *E. coli *PHL628 or *P. fluorescens*. These data were further confirmed by the disc diffusion assay. No inhibition halo surrounding the discs was observed, thereby indicating that the supernatant has no bacteriostatic or bactericidal activity against *E. coli *PHL628 and *P. fluorescens*.

**Figure 4:**
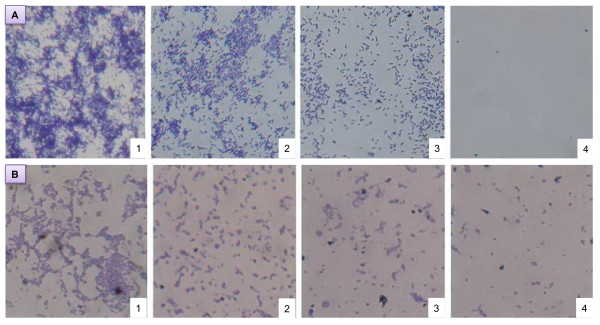
**Microscope observation of biofilm inhibition**. Biofilm inhibition of *E. coli *PHL628 (A) and *Pseudomonas fluorescens *(B) on glass cover slip under a phase-contrast microscope at a magnification of 40X. Bacterial cells were incubated with (1) 1X SP1 supernatant, (2) 2 × SP1 concentrated supernatant, (3) 5 × SP1 concentrated supernatant, (4) 10 × SP1 concentrated supernatant. No difference in biofilm production was observed in the presence of 1X, 2X, 5X and 10X M63K_10 _sterile medium (not shown).

The efficiency of the sponge-associated SP1 supernatant for anti-biofilm activity was evaluated also by microscopic visualization. This approach confirmed that the inhibitory effect of the supernatant on biofilm formation increases with the increase of its concentration. Ten-fold concentrated supernatant completely inhibited biofilm formation by *E. coli *PHL628. Less concentrated supernatant also showed significant reduction of biofilm formation as compared to the control (Figure 4A). Very similar effects were observed with *P. fluorescens *(Figure 4B).

### Inhibitory effect of the supernatant on various strains

**Figure 5:**
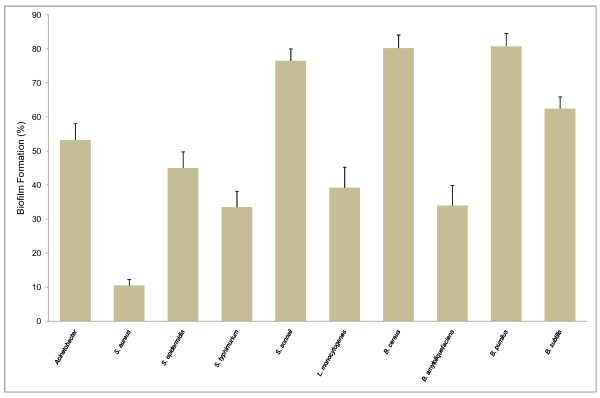
**Inhibitory effect of the SP1 supernatant over a range of Gram-positive and Gram-negative bacteria**. Biofilms of various Gram-positive and Gram-negative bacteria were developed in the presence or absence of the SP1 supernatant (5% V/v) in 96-well microtiter plate. The plate was incubated at 30°C for 36 h, followed by crystal violet staining and spectrophotometric absorbance measurements (OD_570_). The ratio of biofilm absorbance/planktonic absorbance was calculated, and this value used to calculate the "biofilm formation" on the *y axis*. The various Gram-positive and Gram-negative bacteria used in the wells are listed on *X axis*. Bars indicate means ± standard errors for six replicates.

To evaluate further the inhibitory effect of the SP1 supernatant on biofilm development, multiple strains regardless of pathogenicity were tested (Figure 5). Among the strains, 5 out of 10 appeared to be more than 50% inhibited in their biofilm development by the SP1 supernatant. Very interestingly, in the case of *Staphylococcus aureus*, the inhibition was almost 90%. Among the four *Bacillu*s species, *B. amyloliquefaciens *was the most affected one, whereas *B. pumilis *and *B. cereus *were less affected in the inhibition of biofilm development. Not a single strain was stimulated or unaffected in biofilm development by the supernatant.

### Preliminary characterization of the bio-active component of SP1 supernatant

The SP1 cell free supernatant gradually loses its efficiency in decreasing biofilm formation after its pre-treatment at temperatures ranging from 50°C to 80°C. When the supernatant was treated at 50°C, the inhibitory activity towards *E. coli *PHL628 remained 100%, but at 60°C it started to decrease (95%). Treatment at 70°C and 80°C, resulted in 41% and 29% of the anti-biofilm activity respectively. At 90°C the inhibitory activity was completely lost (data not shown).

To preliminarily characterize the mechanism of action of the SP1 supernatant, this was added to bacterial cells together with the quorum sensing signals obtained from two days supernatant of an *E. coli *PHL628 culture in order to understand if there is a competition for the quorum sensing receptor. The use of the two supernatants together had almost the same effect on biofilm inhibition as the SP1 alone (data not shown).

**Figure 6:**
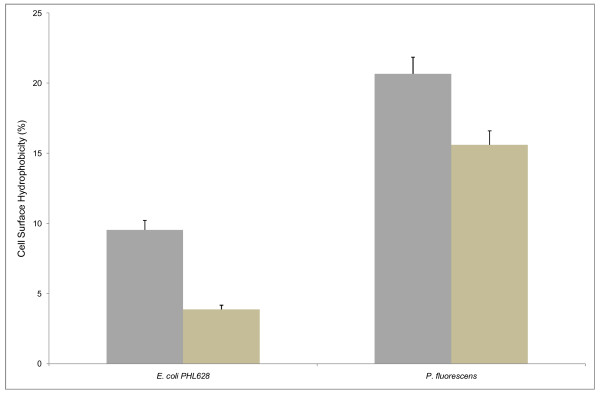
**Cell surface hydrophobicity (CSH) assay for *E. coli *PHL628 and *P. fluorescens***. *E. coli *PHL628 and *P. fluorescens *were grown in minimal medium M63K_10 _and M63, respectively, in the presence (light tan bars) and absence (gray bars) of SP1 supernatant. Bars represent means ± standard errors for six replicates.

To analyze whether inhibition of biofilm production is related to reduced adherence of target cells to surfaces, we tested (see Methods) the effects of SP1 supernatant on the degree of cell surface hydrophobicity of *E. coli *PHL628 and *P. fluorescens*. As shown in Figure 6, the supernatant inhibits significantly the surface hydrophobicity of *E. coli *and to a lesser extent also that of *P. fluorescens*.

### Pre-coating with SP1 supernatant inhibits initial attachment to the abiotic surface

**Figure 7:**
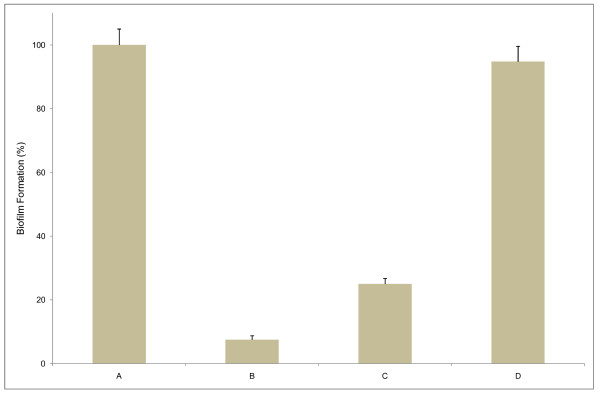
**Pre-coating with the SP1 supernatant reduces attachment during biofilm formation**. Biofilms of *E. coli *PHL628 were developed in 96-well microtiter plates in different conditions: no supernatant (A), wells pre-coated with supernatant (B), supernatant present (C), and supernatant added to pre-formed *E. coli *biofilm (D). The plate was incubated for 36 h, followed by crystal violet staining and spectrophotometric absorbance measurements (OD_570_). The ratio of biofilm absorbance/planktonic absorbance was calculated, and this value is presented as the "biofilm formation" on the *y axis*. Bars represent means ± standard errors for six replicates.

The polysaccharide present in the SP1 supernatant might modify the abiotic surface in such a way that there might be a reduction or inhibition of irreversible attachment of the biofilm forming bacteria to an inanimate object. We tested this hypothesis by analyzing whether there is an effect on biofilm production by *E. coli *PHL628 if the polystyrene wells of the microtiter plate are pre-coated with SP1 supernatant. We observed that after 36 h, while biofilm formation was inhibited by 75% in the un-coated wells and in presence of supernatant, in the pre-coated wells the biofilm assay performed an inhibition of 92.5% (Figure 7). In addition, to evaluate further the mechanism of action in the initial attachment stage of biofilm development, the supernatant was added in the already formed biofilm. The effects were found to be much lower compared to that of the initial addition or pre-coating of the supernatant in the microtiter wells. A possible conclusion of this experiment is that the supernatant modifies the target surface in a way that prevents biofilm formation and that the initial attachment step is most important for biofilms production, at least by the organisms studied in this work.

## Discussion

Marine biota is a potential source for the isolation of novel anti-biofilm compounds [12]. It has been estimated that among all the microbes isolated from marine invertebrates, especially sponge associated, *Bacillus *species are the most frequently found members so far [28]. Therefore the identification, in the present study, of a sponge-associated *Bacillus licheniformis *having anti-biofilm activity is not surprising. Our study demonstrates the occurrence of anti-biofilm activity of a previously uncharacterized polymeric polysaccharide having monomeric structure of galactose-glycerol-phosphate. To our knowledge, no literature has ever reported the finding of such a bioactive compound from marine or other sources.

We found that the polysaccharide is secreted in the culture supernatant by the sponge-associated *B. licheniformis *and its addition to a range of Gram-positive and Gram-negative bacteria results in negative effect on their biofilm development. This broad spectrum of anti-biofilm activity might help *B. licheniformis *during a competitive edge in the marine environment to establish itself on the surface of host sponges and critically influence the development of unique bacterial community.

It has been previously reported that bacterial extracellular polysaccharides can be involved both in biofilm and anti-biofilm activities. For example EPSs from *V. cholera *containing the neutral sugars glucose and galactose are important architectural components of its biofilm [29-31]. On the other hand, EPSs from *E. coli *(group II capsular polysaccharide) [26], *V. vulnificus *(capsular polysaccharide) [32], *P. aeruginosa *(mainly extracellular polysaccharide) [27,33] and marine bacterium *Vibrio *sp.QY101 (exopolysaccharide) [17] display selective or broad spectrum anti-biofilm activity. However, the potentiality of the polysaccharide described in this study over a wide range of pathogenic and non pathogenic organisms suggests that the compound might be a powerful alternative among the previously identified polysaccharides in multispecies biofilm context.

Based on the findings, we hypothesize that our polysaccharide might interfere with the cell-surface influencing cell-cell interactions, which is the pre-requisite for biofilm development [34], or with other steps of biofilm assembling. It has been reported in other cases that polysaccharides can produce anti-adherence effects between microorganisms and surfaces [35]. The *E. coli *group II CPS and exo-polysaccharides of marine *Vibrio *sp. were reported to inhibit biofilm formation not only by weakening cell-surface contacts but also by reducing cell-cell interactions or disrupting the interactions of cell-surfaces and cell-cell [26,17]. In all the previously described polysaccharides having anti-adherence property, highly anionic nature was proposed to be the cause of interference with the adherence of cell-surface and cell-cell [26,17,36]. The *B. licheniformis *compound reported here has also high content of phosphate groups and thus it can be proposed that the electronegative property of the compound might modulate the surface of the tested organism in such a way that there is a reduction or complete inhibition of the attachment of cell-surface or cell-cell.

It might be possible that the compound can modify the physicochemical characteristics and the architecture of the outermost surface of biofilm forming organisms which is the phenomenon observed for some antibiotics [37]. Reduction of cell surface hydrophobicity of *E. coli *PHL628 and *P. fluorescens *clearly indicates the modification of the cell surface, resulting in reduced colonization and thereby significant contribution to anti-biofilm effect. Almost similar results were obtained with coral-associated bacterial extracts for the anti-biofilm activity against *Streptococcus pyogenes *[14].

Anti-biofilm effects were reported to be accompanied in most cases by a loss of cell viability or the presence of quorum sensing analogues. Interestingly, the polysaccharide in the present study is devoid of antibacterial effect, which was demonstrated by the growth curve analysis and disc diffusion test with *E. coli *PHL628 and *P. fluorescens*. An almost similar observation has been reported with the exo-polysaccharide from the marine bacterium *Vibrio *sp. which displayed anti-biofilm nature without decreasing bacterial viability [17]. However, further experiments suggest that the present polysaccharide enhances the planktonic growth of *E. coli *PHL628 in the microtiter plate wells during biofilm production (data not shown). Another interesting phenomenon of the bioactive compound reported here is the absence of competition with the quorum sensing signals presumably present in supernatants of the target biofilm-forming bacteria used in this study. In addition, none of the previously reported quorum sensing competitors is structurally related to the polysaccharide reported here.

In the cover slip experiment, biofilm inhibition was also evidenced and displayed a gradual decrease of biofilm development with the increase of the concentration of the polysaccharide in the culture of *E. coli PHL*628 and *P. fluorescens*. In addition, pre-coating the wells of the polystyrene microtiter plate with the compound also effectively inhibits biofilm formation. To our knowledge, coating with the polysaccharide from sponge-associated bacteria for inhibition of biofilm formation has been reported for the first time here, although there are some reports on the use of pre-coating surfaces with different surfactants and enzymes [38-41].

In conclusion, the polysaccharide isolated from sponge-associated *B. licheniformis *has several features that provide a tool for better exploration of novel anti-biofilm compounds. Inhibiting biofilm formation of a wide range of bacteria without affecting their growth represents a special feature of the polysaccharide described in this report. This characteristic has already been described for other polysaccharides in a few very recent articles [40-42]. Further research on such surface-active compounds might help developing new classes of anti-biofilm molecules with broad spectrum activity and more in general will allow to explore new functions of bacterial polysaccharides in the environment.

## Methods

### Isolation of bacterial strains

The bacterial strains used in this study were initially obtained from an orange-colored sponge, *Spongia officinalis*, collected from Mazara del Vallo (Sicilia, Italy), from a depth of 10 m. The sponge sample was transferred soon after collection to a sterile falcon tube and transported under frozen condition to the laboratory for the isolation of associated microbes. The sponge was then mixed with sterile saline water and vortexed. A small fraction of the liquid was serially diluted up to 10^-3 ^dilutions and then spread on plates of Tryptone Yeast agar (TY). The plates were incubated at 37°C for 2 days till growth of colonies was observed. Single bacterial colonies were isolated on the basis of distinct colony morphologies from the TY plates. Isolates were maintained on TY agar plates at 4°C until use.

### Supernatant preparation

The isolated bacteria were sub-cultured on M63 (minimal medium) agar plates and incubated at 37°C for two days. A loopful of the bacterial culture from each plate was inoculated into M63 broth (in duplicate), incubated at 37°C for 24 h and then centrifuged at 7000× g for 20 minutes to separate the cell pellets from the fermentation medium. The supernatants were filtered through 0.2 μm-pore-size Minisart filters (Sartorius, Hannover, Germany). To ensure that no cells were present in the filtrates, 100 μl were spread onto TY agar plates, and 200 μl were inoculated in separate wells in the microtiter plate.

### Screening for bioactive metabolites for biofilm inhibition

Filtered supernatants from the marine sponge-associated isolates were used to perform the assay for biofilm formation. The method used was a modified version of that described by Djordjevic *et al*. [43]. Overnight cultures of *E. coli *PHL628 strain grown at 37°C in M63K_10 _broth (M63 broth with kanamycine, 10 μg ml^-1^), were refreshed in M63K_10 _broth and incubated again at 37°C for 5 to 6 h. 200 μl of inocula were introduced in the 96 well polystyrene microtiter plate with an initial turbidity at 600 nm of 0.05 in presence of the filtered supernatants from the different marine sponge associated isolates. The microtiter plate was then left at 30°C for 36 h in static condition.

To correlate biofilm formation with planktonic growth in each well, the planktonic cell fraction was transferred to a new microtiter plate and the OD_570 _was measured using a microtiter plate reader (*Multiscan Spectrum, Thermo Electron Corporation*). To assay the biofilm formation, the remaining medium in the incubated microtiter plate was removed and the wells were washed five times with sterile distilled water to remove loosely associated bacteria. Plates were air-dried for 45 min and each well was stained with 200 μl of 1% crystal violet solution for 45 min. After staining, plates were washed with sterile distilled water five times. The quantitative analysis of biofilm production was performed by adding 200 μl of ethanol-acetone solution (4:1) to de-stain the wells. The level (OD) of the crystal violet present in the de-staining solution was measured at 570 nm. Normalized biofilm was calculated by dividing the OD values of total biofilm by that of planktonic growth. Six replicate wells were made for each experimental parameter and each data point was averaged from these six.

### Identification and purification of anti-biofilm compound

144 ml of cell free bacterial broth cultures were extensively dialyzed against water for two days, using a membrane tube of 12000-14000 cut-off; this procedure allowed us to remove the large amount of glycerol in the bacterial broth as confirmed by ^1^H- ^13^C-NMR experiments recorded on lyophilized broth before and after dialysis; the inner dialysate (25 mg) was fractionated by gel filtration on Sepharose CL6B, eluting with water. Column fractions were analyzed and pooled according to the presence of saccharidic compounds, proteins and nucleic acids. Fractions were tested for carbohydrate qualitatively by spot test on TLC sprayed with *α *-naphthol and quantitatively by the Dubois method [44]. Protein content was estimated grossly by spot test on TLC sprayed with ninhydrin and by reading the column fractions absorbance at 280 nm. The active fractions were tested by the Bio-Rad Protein System, with the bovine serum albumin as standard [45]. Finally, the presence of nucleic acids was checked by analysis of fractions absorbance at 260 nm. Furthermore, the grouped fractions were investigated by ^1^H-NMR spectroscopy. ^1^H and ^13^C NMR spectra, were recorded at 600.13 MHz on a BrukerDRX-600 spectrometer, equipped with a TCI CryoProbeTM, fitted with a gradient along the Z-axis, whereas for ^31^P-NMR spectra a Bruker DRX-400 spectrometer was used.

The gel filtration fractions were tested for anti-biofilm activity and the active fraction resulted positive to carbohydrate tests; this latter was a homogenous polysaccharide (6.6 mg) material. Preliminary spectroscopic investigations indicated the presence of a compound with a simple primary structure; the molecular mass of polysaccharidic molecule was estimated by gel filtration on a Sepharose CL6B which had previously been calibrated by dextrans (with a Mw from 10 to 2000 kDa). It's worthy to notice that some resonances in ^13^C NMR spectrum (78.32, 70.76, 65.63, 67.15 ppm) were split; this suggested the presence of ^31^P (J_C-P _from 4 to 9 Hz, see table 1) and its position into the polysaccharide repeating unit.

The phosphate substitution was confirmed by recording a ^31^P-NMR spectrum; it showed a single resonance at 1.269 ppm [46].

The GC-MS analysis of the high-molecular-weight polymer was carried out on an ion-trap MS instrument in EI mode (70eV) (Thermo, Polaris Q) connected with a GC system (Thermo, GCQ) by a 5% diphenyl (30 m × 0.25 mm × 0.25 um) column using helium as gas carrier. Nuclear Overhauser enhancement spectroscopy experiments (NOESY) were acquired using a mixing time of 100 and 150 ms. Total correlation spectroscopy experiments (TOCSY) were performed with a spinlock time of 68 ms.

Heteronuclear single quantum coherence (HSQC) and heteronuclear multiple bond correlation (HMBC) experiments were measured in the ^1^H-detected mode via single quantum coherence with proton decoupling in the ^13^C domain. Experiments were carried out in the phase-sensitive mode and 50 and 83 ms delays were used for the evolution of long-range connectivities in the HMBC experiment. The 2D ^1^H-^31^P HSQC experiment was recorded setting the coupling constants at 10 and 20 Hz.

### Growth curve analysis

The effect of the bioactive compound on the planktonic culture was checked by growth curve analysis on both *E. coli *PHL628 and *Pseudomonas fluorescens*. The supernatant of the isolate was added to a conical flask containing 50 ml of M63 broth, to which a 1% inoculum from the overnight culture was added. The flask was incubated at 37°C. Growth medium with the addition of bacterial inoculum and without the addition of the supernatant was used as a control. OD values were recorded for up to 24 h at 1-h intervals.

### Antibacterial activity by disk diffusion assay

Antimicrobial activity of the supernatant was assayed by the disc diffusion susceptibility test (Clinical and Laboratory Standards Institute, 2006). The disc diffusion test was performed in Muller-Hinton agar (MHA). Overnight cultures of *E. coli *PHL628 and *P. fluorescens *were subcultured in TY broth until a turbidity of 0.5 McFarland (1 × 10^8 ^CFU ml^-1^) was reached. Using a sterile cotton swab, the culture was uniformly spread over the surface of the agar plate. Absorption of excess moisture was allowed to occur for 10 minutes. Then sterile discs with a diameter of 10 mm were placed over the swabbed plates and 50 μl of the extracts were loaded on to the disc. MHA plates were then incubated at 37°C and the zone of inhibition was measured after 24 h.

### Microscopic techniques

For visualization of the effect of the sponge-associated bacterial supernatant against the biofilm forming *E. coli *PHL628 and *Pseudomonas fluorescens*, the biofilms were allowed to grow on glass pieces (1 × 1 cm) placed in 6-well cell culture plate (*Greiner Bio-one, Frickenhausen, Germany*). The supernatant at concentrations ranging from 1 to 10 times was added in M63K_10 _(for *E. coli *PHL628) and M63 broth (for *P. fluorescens*) containing the bacterial suspension of 0.05 O.D. at 600 nm. The wells without supernatant were used as control.

The plate was incubated for 36 h at 30°C in static condition. After incubation, each well was treated with 0.4% crystal violet for 45 minutes. Stained glass pieces were placed on slides with the bio-film pointing up and were inspected by light microscopy at magnifications of ×40. Visible bio-films were documented with an attached digital camera (*Nikon Eclipse Ti 100*).

### Anti-biofilm effect on various strains and growth conditions

Some laboratory strains such as *Acinetobacter*, *Staphylococcus aureus*, *Staphylococcus epidermidis*, *Salmonella typhimurium*, *Shigella sonneii*, *Listeria monocytogenes*, *Bacillus cereus*, *Bacillus amyloliquefaciens*, *Bacillus pumilus *and *Bacillus subtilis *were selected. All strains were grown in Tryptone Soya Broth (TSB) (Sigma) supplemented with 0.25% glucose and the same medium was used during the biofilm assay in the presence of SP1 supernatant.

### Competitiveness between quorum sensing factors and bioactive compounds

For this experiment the *E. coli *PHL628 supernatant was prepared by using the same conditions as for that of the sponge-isolated strain. Equal volumes of the two supernatants were added either in combination or alone in the microtiter plate containing a culture of *E. coli *PHL628 at an initial turbidity of 0.05 at 600 nm and biofilm formation was measured as described above. Each result was an average of at least 6 replicate wells.

### Pre-coating of microtiter plate

Wells were treated with 200 μl of the *B. licheniformis *supernatant for 24 h and then the un-adsorbed supernatant was withdrawn from the wells. Such pre-coated wells were inoculated with *E. coli *PHL628 cultures having an OD of 0.05 at 600 nm. In another set of wells that were not coated with the supernatant, the fresh culture of *E. coli PHL628 *having the same density mentioned above were added together with the supernatant (5% v/v). The microtiter plate was then incubated for 36 h in static conditions and biofilm formation was estimated. The control experiments were carried out in wells that were not pre-coated or initially added with the supernatant. Each result was an average of at least 6 replicate wells and three independent experiments.

In a parallel microtiter plate, the supernatant was added to the 36-h biofilm culture in the microtiter plate and was then left at 30°C in static conditions for another 24 h. The experiment was repeated six times to validate the results statistically.

### Microbial cell surface hydrophobicity (CSH) assay

Hydrophobicity of the culture of *E. coli *PHL628 and *P. fluorescens *were determined by using MATH (microbial adhesion to hydrocarbons) assay as a measure of their adherence to the hydrophobic hydrocarbon (toluene) following the procedure described by Courtney *et al*. 2009 [47]. Briefly, 1 ml of bacterial culture (OD530 nm = 1.0) was placed into glass tubes and 100 μl of toluene along with the supernatant (5% v/v) was added. The mixtures were vigorously vortexed for 2 min, incubated 10-min at room temperature to allow phase separation, then the OD530 nm of the lower, aqueous phase was recorded. Controls consisted of cells alone incubated with toluene. The percentage of hydrophobicity was calculated according to the formula: % hydrophobicity = [1-(OD530 nm after vortexing/OD530 nm before vortexing)]×100.

## Competing interests

The authors declare that they have no competing interests.

## Authors' contributions

MV planned the work that led to the manuscript; SMAS produced and analyzed the experimental data; AZ, AC and MDF participated in the interpretation of the results; MV, SMAS and MDF wrote the paper; EM, LC and AT performed the chemical characterization of the bioactive compound. All authors read and approved the final manuscript.
